# Supplementary material for: Structural rearrangements underlying the activation of STIM1 by ER calcium depletion
Source: bioRxiv. 2026 Jan 20:2026.01.16.700022. Preprint. [Version 1] doi: 10.64898/2026.01.16.700022 (PMC12871627; doi:10.64898/2026.01.16.700022)
Supplement: 1 [file NIHPP2026.01.16.700022V1-supplement-1.pdf]

bioRxiv preprint doi: https://doi.org/10.64898/2026.01.16.700022; this version posted January 20, 2026. The copyright holder for this preprint (which was not certified by peer review) is the author/funder, who has granted bioRxiv a license to display the preprint in perpetuity. It is made available under aCC-BY 4.0 International license.

SUPPLEMENTARY FIGURES

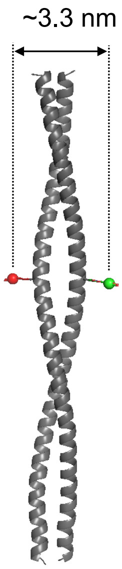

PDB:1D7M
Cortexillin I coiled-coil

Figure S1. Dye-dye distance simulation in cortexillin I coiled-coil structure. Dye positions were simulated using a CNS-based approach based on the cortexillin I coiled-coil structure (PDB: 1D7M). The inter-dye distance across the two symmetric helices of the coiled-coil was estimated to be approximately 3.3 nm.

bioRxiv preprint doi: https://doi.org/10.64898/2026.01.16.700022; this version posted January 20, 2026. The copyright holder for this preprint (which was not certified by peer review) is the author/funder, who has granted bioRxiv a license to display the preprint in perpetuity. It is made available under aCC-BY 4.0 International license.

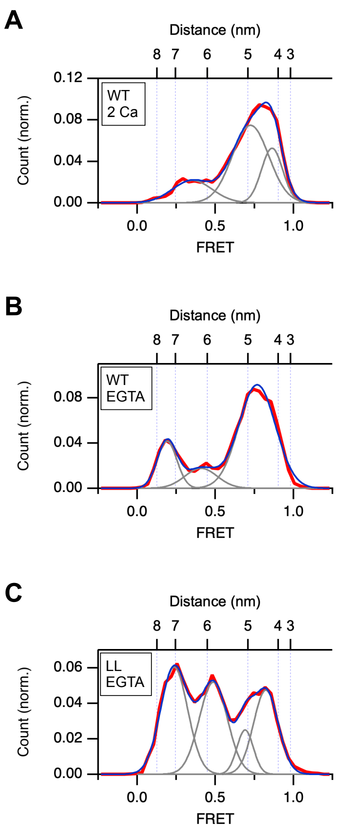

Figure S2. Effects of the N234L/S237L double mutation (LL) on the CC1α2/3 hairpin. CC1α2-CC1α3 (291:325) FRET histograms are shown with fitted Gaussian curves (gray) and their sum (blue) superimposed on the data (red). (*A*) WT STIM1 in 2 mM Ca^{2+} (n=202). Fit parameters (peak FRET and fractional area): 0.35 (18%), 0.73 (58%), 0.86 (24%). (*B*) WT STIM1 in 0.5 mM EGTA (n=246). Fit parameters (peak FRET and fractional area): 0.19 (18%), 0.42 (12%), 0.75 (60%), 0.88 (10%). (*C*) STIM1-LL in 0.5 mM EGTA (n=219). Fit parameters (peak FRET and fractional area): 0.24 (35%), 0.49 (31%), 0.69 (9%), 0.82 (25%). The significant increase of low FRET fraction in STIM1-LL indicates the unfolding and extension of the CC1α2/3 hairpin, consistent with the formation of the CC1 coiled-coil.

bioRxiv preprint doi: https://doi.org/10.64898/2026.01.16.700022; this version posted January 20, 2026. The copyright holder for this preprint (which was not certified by peer review) is the author/funder, who has granted bioRxiv a license to display the preprint in perpetuity. It is made available under aCC-BY 4.0 International license.

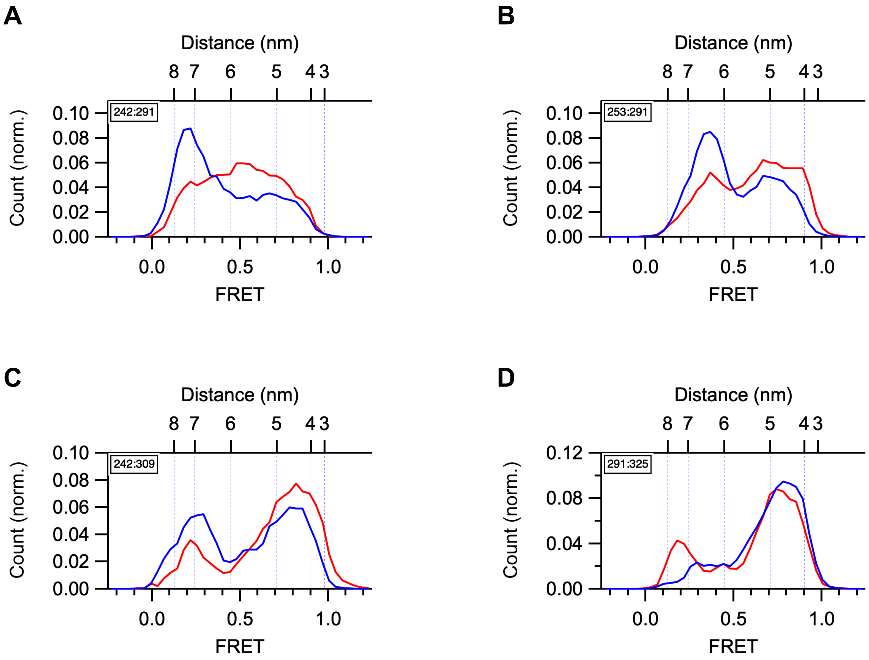

Figure S3. Comparison of intrasubunit distances in CC1 in Ca^{2+} and EGTA. FRET histograms are shown for 2 mM Ca^{2+} (blue) and 0.5 mM EGTA (red) with numbers of molecules (in Ca^{2+}, in EGTA). (*A*) 242:291 (n=192, 189). (*B*) 253:291 (n=254, 275). (*C*) 242:309 (n=82, 158). (*D*) 291:325 (n=202, 210).

bioRxiv preprint doi: https://doi.org/10.64898/2026.01.16.700022; this version posted January 20, 2026. The copyright holder for this preprint (which was not certified by peer review) is the author/funder, who has granted bioRxiv a license to display the preprint in perpetuity. It is made available under aCC-BY 4.0 International license.

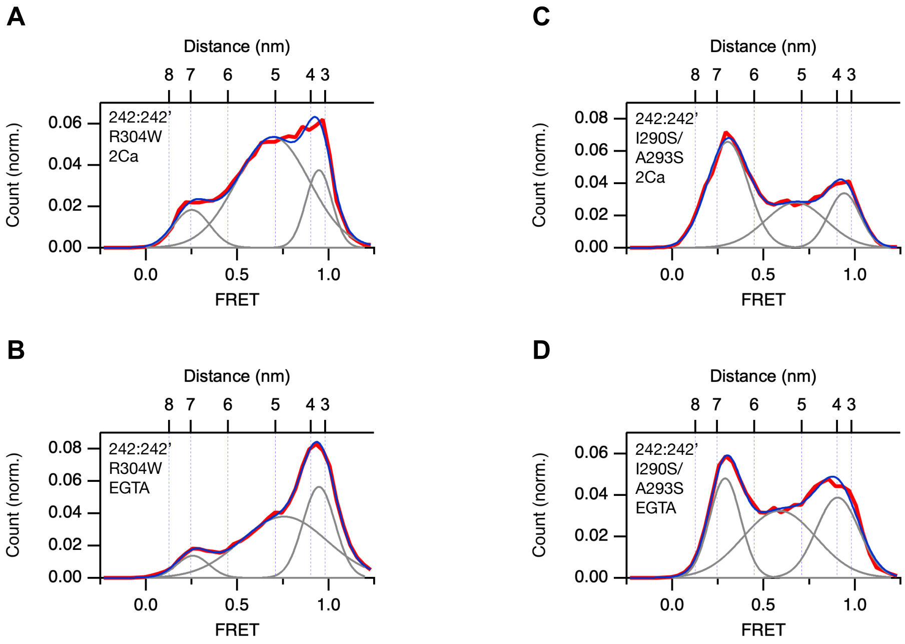

Figure S4. Destabilizing the 3HB promotes transitions to the fully extended coiled-coil state. smFRET histograms are shown with fitted Gaussian curves (gray) and their sum (blue) superimposed on the data (red). (*A*) CC1α1-CC1α1' FRET histogram of STIM1-R304W in 2 mM Ca^{2+} (242:242'; n=225). Fit parameters (peak FRET and fractional area): 0.25 (12%), 0.70 (70%), 0.95 (18%). (*B*) CC1α1-CC1α1' FRET histogram of STIM1-R304W in 0.5 mM EGTA (242:242'; n=356). Fit parameters (peak FRET and fractional area): 0.26 (8%), 0.76 (59%), 0.95 (33%). (*C*) CC1α1-CC1α1' FRET histogram of I290S/A293S-STIM1 in 2 mM Ca^{2+} (242:242'; n=316). Fit parameters (peak FRET and fractional area): 0.30 (49%), 0.68 (31%), 0.94 (20%). (*D*) CC1α1-CC1α1' FRET histogram of STIM1-I290S/A293S in 0.5 mM EGTA (242:242'; n=434). Fit parameters (peak FRET and fractional area): 0.29 (27%), 0.59 (43%), 0.91 (30%).

bioRxiv preprint doi: https://doi.org/10.64898/2026.01.16.700022; this version posted January 20, 2026. The copyright holder for this preprint (which was not certified by peer review) is the author/funder, who has granted bioRxiv a license to display the preprint in perpetuity. It is made available under aCC-BY 4.0 International license.

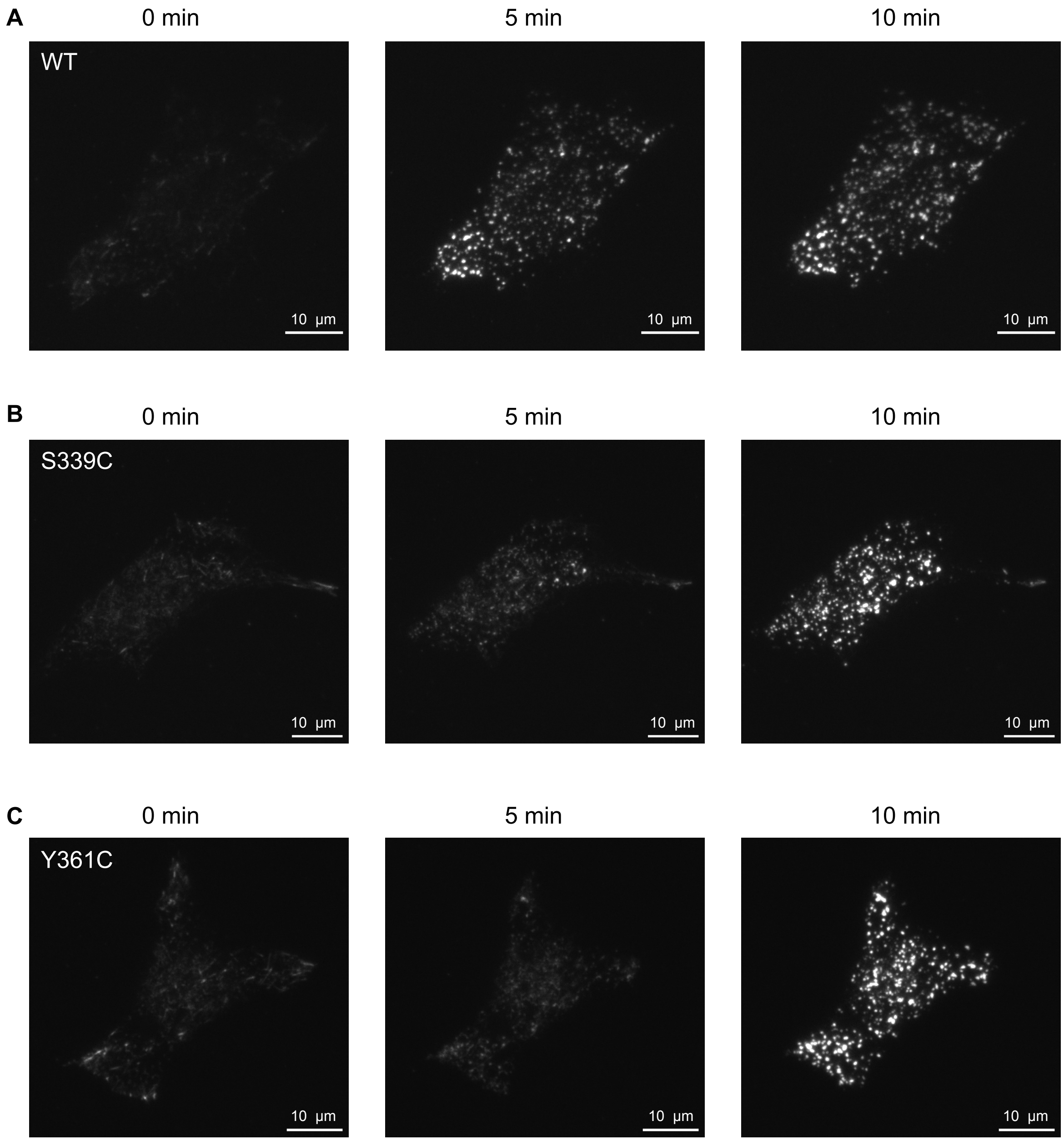

Figure S5. Kinetics of STIM1 puncta formation are slowed by crosslinking S339C or Y361C. Representative TIRF images of mCh-STIM1 puncta at different time points for STIM1-WT (*A*), STIM1-S339C (*B*), and STIM1-Y361C (*C*), taken from the experiments shown in **Figs. 4 and 7**.

bioRxiv preprint doi: https://doi.org/10.64898/2026.01.16.700022; this version posted January 20, 2026. The copyright holder for this preprint (which was not certified by peer review) is the author/funder, who has granted bioRxiv a license to display the preprint in perpetuity. It is made available under aCC-BY 4.0 International license.

SUPPLEMENTARY TABLES

Dye pair	Peak FRET	Number of molecules	Distance from FRET (nm)	Distance from CAD crystal structure (nm)	Distance from AlphaFold2 (nm)
337:337’	0.78	183	4.70	NA	4.08
349:349’	0.84	221	4.40	4.29	4.31
378:378’	0.93	226	3.77	3.51	3.41
431:431’	0.78	204	4.70	4.69	4.85
349:431	0.86	223	4.29	3.88	4.12
349:431’	0.97	232	3.25	3.17	2.74

Table S1. Comparison of FRET-derived distances in 2 mM Ca^{2+} with the CAD crystal structure and the AlphaFold2 model of STIM1. For each dye pair, the predominant smFRET value from the STIM1-WT amplitude histograms is listed and used to calculate a distance. The number of single molecule traces used to construct each histogram is indicated. Inter-dye distances from the CAD crystal structure (3TEQ.pdb) and AlphaFold2 model (Qiu and Lewis, 2025) were measured as described in Methods. NA indicates that the residues are absent in the CAD crystal structure.
